# The emergence of *mecC* methicillin-resistant *Staphylococcus aureus*

**DOI:** 10.1016/j.tim.2013.11.003

**Published:** 2014-01

**Authors:** Gavin K. Paterson, Ewan M. Harrison, Mark A. Holmes

**Affiliations:** Department of Veterinary Medicine, University of Cambridge, Cambridge CB3 0ES, UK

**Keywords:** methicillin-resistant *Staphylococcus aureus* (MRSA), *mecC*, zoonosis, antibiotic resistance, molecular epidemiology, genome sequencing

## Abstract

The report of methicillin-resistant *Staphylococcus aureus* (MRSA) encoding a divergent *mecA* gene in 2011 was highly significant. This homologue, designated *mecC*, poses diagnostic problems with the potential to be misdiagnosed as methicillin-sensitive *S*. *aureus*, with important potential consequences for individual patients and for the surveillance of MRSA. *mecC* MRSA have now been reported from 13 European countries and have been isolated from 14 different host species, with evidence of a recent increase in Denmark. The emergence of *mecC* MRSA is a topic of interest to human and veterinary microbiology, and we consider it timely to review here its discovery and subsequent investigation.

## Highlights

• A novel *mecA* homologue, *mecC*, confers methicillin resistance to methicillin-resistant *Staphylococcus aureus* (MRSA).
• *mecC* MRSA produce negative results with common diagnostic tools.
• Reported from throughout Western Europe. Recent increase in Denmark.
• Found in a range of host species and may pose a zoonotic risk to humans.

## *Staphylococcus aureus* and MRSA in humans and animals

*S. aureus* is a prominent human pathogen that can cause a diverse range of diseases ranging from relatively minor skin infections to serious and life-threatening infections such as endocarditis, pneumonia, and sepsis. Its impact is enhanced by the development of antibiotic resistance, most notably methicillin-resistant *S*. *aureus* (MRSA) that is resistant to virtually all β-lactam antibiotics. Although originally regarded as a nosocomial pathogen (hospital-associated MRSA or HA-MRSA), MRSA infections among previously healthy individuals in the community, without links to healthcare settings, emerged in the 1990s and are referred to as community-associated MRSA (CA-MRSA). For the most part HA-MRSA and CA-MRSA involve different lineages, but these distinctions are not absolute, and transfer of strains between these settings is increasingly recognised. In addition to its importance as a human pathogen, *S*. *aureus* [1], including MRSA [2,3], can colonise and infect a wide range of host species including livestock, wildlife, and companion animals, with bovine mastitis among dairy cattle, lameness in poultry, and severe and lethal infections in farmed rabbits being particularly significant in terms of economic impact. MRSA in animals is not only important from an animal welfare and economic perspective but can act as a reservoir for zoonotic infection of humans. In particular, multilocus sequence type clonal complex (CC)398 is abundant among pigs and other livestock in mainland Europe, and infection of humans in close contact with these animals has led to the recognition of a third epidemiological form of MRSA, livestock-associated MRSA (LA-MRSA) [4].

## Mechanism of methicillin-resistance in MRSA and its diagnostic detection

Although methicillin is no longer produced, the name MRSA has persisted and can be regarded as referring to resistance to virtually all β-lactam antibiotics. Susceptibility testing now typically uses oxacillin and/or cefoxitin. β-Lactams bind to the penicillin-binding proteins (PBP) essential for cell wall biosynthesis and inhibit peptidoglycan crosslink formation, leading to bacterial cell lysis. Resistance to β-lactams in MRSA is conferred by the acquisition of a mobile genetic element, the staphylococcal cassette chromosome (SCC*mec*) carrying the *mecA* gene which encodes an altered PBP – PBP2a/PBP2′ – which has reduced affinity for β-lactam antibiotics. As a result, cell wall biosynthesis in MRSA strains continues even in the presence of otherwise inhibitory levels of β-lactam antibiotics. The detection and diagnosis of MRSA in the clinical microbiology setting is very important both for informing the appropriate treatment of individual patients and also for the surveillance of MRSA. The gold standard for confirmation of MRSA is regarded as the molecular detection of either *mecA*, typically by PCR, or of PBP2a/PBP2′, usually by antibody detection with commercially available slide agglutination assays. Crucial to the reliability of these assays is the fact that *mecA* and PBP2a/PBP2′ are both highly conserved among MRSA isolates.

## Discovery of *mecC* MRSA: genome sequencing to identify a novel resistance gene

**Figure 1:**
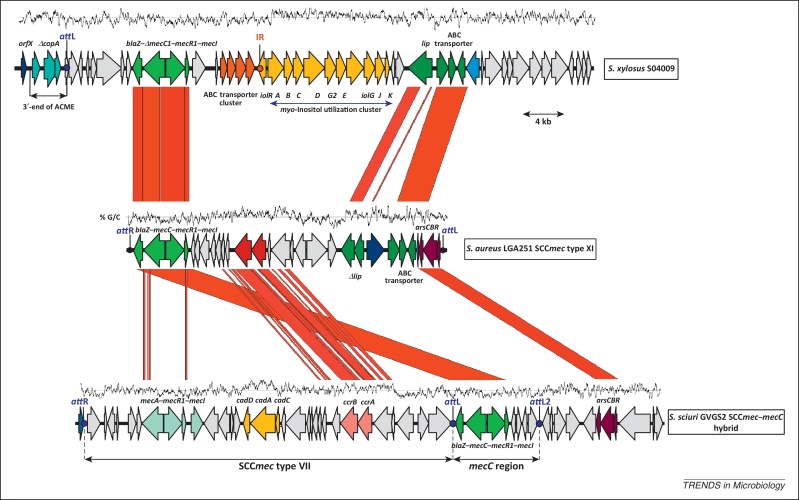
Comparison of the *mecC1* region in *Staphylococcus xylosus* strain S04009 [38] (EMBL accession number HE993884), SCC*mec* type XI in *Staphylococcus aureus* LGA251 [6] (EMBL FR821779), and a hybrid SCC*mec*–*mecC* in *S*. *sciuri* strain GVGS2 [39] (EMBL HG515014). Areas in red show regions conserved between the two sequences; homologous coding sequences are marked in the same colour. Blue and red dots indicate the SCC*mec* attachment sites (*att*L and *att*R) and inverted repeats (IR), respectively. The %G/C content of the region is shown above each genome schematic. Abbreviations: ABC, ATP-binding cassette; ACME, arginine catabolic mobile element; SCC*mec*, staphylococcal cassette chromosome.

An epidemiological study of bovine mastitis [5] led to the isolation in 2007 of a *S*. *aureus* isolate, LGA251, from a bulk tank milk sample in southwest England which was phenotypically MRSA (i.e., resistant to oxacillin and cefoxitin). At that time this in itself was immediately significant because it represented the first detection of MRSA in the UK dairy herd. However, confirmatory tests for the *mecA* gene and PBP2a/2′ were repeatedly negative [6]. Genome sequencing of LGA251 at the Wellcome Trust Sanger Institute revealed that the strain carried a novel *mecA* homologue, initially termed *mecA*_LGA251_, which was only ∼69% identical to conventional *mecA* at the DNA level, and the encoded PBP2a/2′ was ∼63% identical at the amino acid level [6]. This explained the resistance of LGA251 and why it produced negative results by *mecA* PCR and PBP2a/2′ slide agglutination. A retrospective search of isolate collections in the UK and Denmark identified a further 65 isolates positive for *mecA*_LGA251_ isolated not only from dairy cattle but also from humans, including the earliest known isolate, a Danish blood isolate from 1975 [6]. In consequence, although *mecA*_LGA251_ MRSA has only recently been recognised, it may have been causing human infections for over 35 years. These *mecA*_LGA251_ MRSA isolates belonged predominantly to CC130 and ST425 [6]. Similarly to conventional *mecA*, *mec*A_LGA251_ is located within a SCC*mec* element inserted into the 3′ region of *orfX* (Figure 1). The LGA251 SCC*mec* was also novel; in other words, it had divergent *ccrA* and *ccrB* recombinases (belonging to the *ccrA1* and *ccrB3* groups and representing a novel combination of recombinase groups designated type 8 *ccr*), divergent *mecA* regulatory genes (*mecI*/*mecR)*, and the absence of one of the three joining regions (J3) that are normally present [6]. The SCC*mec* sequence from LGA251 was submitted to the Working Group on the Classification of SCC and given the designation type XI SCC*mec* in November 2009. *mecA*_LGA251_ was itself subsequently renamed *mecC* in 2012 [7]. *mecC* was chosen because an additional divergent homologue of *mecA*, distinct from *mecA*_LGA251_, had already been described in *Macrococcus caseolyticus* [8] and was designated *mecB* [7]. Published at the same time as the UK and Danish report [6], work in the Republic of Ireland independently described *mecC* and type XI SCC*mec* in human MRSA strains isolated in 2010 and belonging to CC130 [9].

## Functional characterisation of *mecC*-encoded PBP2a

The function of the *mecC*-encoded PBP2a/2′ and its role in β-lactam resistance was formally demonstrated by the work of Kim *et al*. which also highlighted noteworthy differences in the properties of the *mecA* and *mecC*-encoded proteins [10]. Although the detection of *mecC*-encoded PBP2a in LGA251 was problematic, most likely due to low expression levels resulting from *mecI*/*mecR*, inducible expression of *mecC* in a methicillin-sensitive *S. aureus* (MSSA) strain conferred high minimum inhibitory concentration (MIC) values against a range of β-lactams [10]. Recombinant PBP2a_*mecC*_ protein was bound by β-lactams but showed higher affinity for oxacillin compared to cefoxitin, whereas PBP2a_*mecA*_ showed less preference. The two proteins also displayed differences in their thermostabilty and temperature optima, with PBP2a_*mecC*_ appearing to be less stable at 37 °C than PBP2a_*mecA*_. Interestingly, PBP2a_*mecC*_ did not require the presence of the native PBP2 to confer high-level oxacillin resistance. This is in contrast to PBP2a_*mecA*_ for which high-level oxacillin resistance requires the presence of native PBP2 to provide transglycosylase activity lacking in PBP2a_*mecA*_. Because PBP2a_*mecC*_ also appears to lack transglycosylase activity, high-level oxacillin resistance conferred by *mecC* is likely to involve collaboration between PBP2a_*mecC*_ and one of the other monofunctional glycotransferases that are known to be induced in *S*. *aureus* when PBP2 is inhibited [10]. Although this characterisation confirms the function of *mecC*-encoded PBP2a as a transpeptidase, and its role in methicillin resistance, there are important differences in the behaviour of the proteins encoded by *mecC* and *mecA*. The structural and evolutionary bases for these distinctions are not yet clear.

## The issue of *mecC* MRSA detection

Although there are obviously differences in biochemistry between *mecA* and *mecC*-encoded PBP2a, *mecC* nonetheless confers methicillin resistance, and such strains need to be identified correctly as MRSA in diagnostic laboratories. Where laboratories are performing antimicrobial susceptibility testing, *mecC* MRSA will likely be correctly identified as MRSA. Importantly, cefoxitin has been found to be more reliable than oxacillin in disc diffusion, broth microdilution, and agar dilution assays [11]. However, significant differences in the reliability of agars from different manufacturers have been described [11].

Similarly, *mecC* MRSA produce a distinctive antibiotic susceptibility profile compared to *mecA* MRSA when assayed using the automated Vitek 2 system from BioMérieux [12]. Where both oxacillin and cefoxitin are included, *mecA* MRSA, as might be expected, typically display resistance to both. By contrast, the majority of *mecC* MRSA show resistance to cefoxitin, and are therefore reported as MRSA, but however show susceptibility to oxacillin. Testing of a panel of 896 *S. aureus* isolates (comprising *mecA* MRSA, *mecC* MRSA, and *mec*-negative MSSA) found that this oxacillin-sensitive/cefoxitin-resistant profile had a sensitivity of 88.7% and a specificity of 99.5% for the identification of *mecC* MRSA isolates from MSSA and *mecA* MRSA [12]. This profile therefore provides a zero-cost screening method for identification of *mecC*-positive MRSA strains in the many clinical laboratories already using Vitek 2, although subsequent PCR would be needed to confirm *mecC* status. The performance of other automated systems for the detection of *mecC* MRSA resistance has not been fully tested and reported.

The differences in oxacillin and cefoxitin sensitivities displayed by *mecC* MRSA isolates are consistent with the findings of Kim *et al*. discussed above, demonstrating that the *mecC*-encoded PBP2a, unlike the *mecA*-encoded counterpart, has a higher relative affinity for oxacillin than for cefoxitin, leading to higher levels of resistance to cefoxitin than to oxacillin [10].

*mecC* MRSA appear to grow reliably on commercial chromogenic agar plates designed to identify MRSA, although there are indications that some MRSA agars may perform better than others for the recovery of *mecC* MRSA [13]. *mecC* MRSA typically have lower MICs to oxacillin and cefoxitin than their *mecA* counterparts, and this may affect their recovery on selective agars.

One major problem is where molecular detection of *mecA* is used to identify or confirm MRSA. Laboratories using this approach, most often PCR, will need to consider incorporating universal *mec* gene primers able to amplify both *mecA* and *mecC* or the addition of *mecC*-specific primers. This latter option has the benefit of differentiating *mecC* MRSA, thereby facilitating their surveillance and the isolation of strains for further characterisation. Various modified PCR assays have been developed to detect and/or differentiate *mecC* MRSA [14–16], and many commercial PCR-based assays are being, or have been, modified to include *mecC* detection [14,17]. Commercial slide agglutination assays for *mecA*-encoded PBP2a will also misidentify *mecC* MRSA as being methicillin-susceptible. These tests may be modified in due course to detect *mecC* MRSA, but currently the use of commercial slide agglutination assays alone will produce false-negative results for these strains. Strains found to be phenotypically resistant but *mecA* and/or PBP2a-negative are potentially *mecC* MRSA, and *mecC* PCR would be warranted to confirm this. *mec* gene*-*negative MRSA have also been reported [18].

In summary, *mecC* MRSA pose a potential diagnostic loophole which clinical microbiology laboratories should be aware of and which will require validation of testing approaches to ensure that *mecC* MRSA are correctly identified as MRSA. Statistically robust, formal studies are needed to validate the diverse MRSA susceptibility testing regimes for their correct identification of *mecC* MRSA as methicillin-resistant, even if only to confirm that current methods are sufficient.

## Epidemiology of *mecC* MRSA in humans and animals

Following the original discoveries of *mecC* MRSA in the UK, Denmark, and the Republic of Ireland such strains, both human and animal origin, were rapidly identified in a further 10 Western European countries (Table 1). In many cases these reports represent small numbers of isolates identified by opportunistic sampling; for example, retrospective testing of previously identified atypical MRSA isolates. From these data it is unclear how common *mecC* MRSA truly are. In Denmark, however, where reporting of human MRSA is mandatory and extensive strain collections are maintained, the prevalence of *mecC* MRSA among all MRSA was found to be 1.9% in 2010, increasing to 2.8% in 2011 [19]. Further evidence supporting a recent increase is that very few Danish *S. aureus* isolates collected prior to 2003 were found to be *mecC* MRSA [19]. By comparison, large-scale collection and characterisation of human MRSA in Germany found only two *mecC* MRSA isolates among 3207 MRSA isolates (prevalence 0.06%), with no indication of a change in prevalence between 2004/05 and 2010/11 [20]. In the UK, a study in England during 2011–2012 surveyed 335 sequential MRSA isolates from individual patients collected from each of six clinical microbiological laboratories, and found a prevalence rate for *mecC* MRSA of 0.45% (nine *mecC* MRSA isolates from a total of 2010 MRSA isolates collected) [21]. The screening of 565 *S. aureus* isolates collected between 2005 and 2011 in western Switzerland did not identify any *mecC* MRSA isolates, suggesting that these are also rare in that country [22]. *mecC* MRSA has yet to be reported from outside Western Europe, and a small survey of US service personnel injured during deployment in Iraq and Afghanistan and transitioned through Germany *en route* to the USA found no *mecC* MRSA among 102 MRSA isolates [23]. *mecC* MRSA currently appears to be uncommon in humans, but there are interesting geographical differences in prevalence; the recent increase in Denmark highlights the need to monitor *mecC* MRSA.

Although a number of multilocus sequence types have been found among *mecC* MRSA isolates, two major lineages are responsible for the vast majority of isolates to date: CC130, which seems to predominate, and ST425. Among these lineages a large number of *spa*-types are represented (Table 1), with t843, associated with CC130, being the most common. *mecC*-negative ST425 have also been reported [24], as have CC130 MSSA, although the *mec* gene status of the latter was not confirmed [25].

*mecC* MRSA have been found in a wide range of other host species encompassing livestock, wildlife, and companion animals from many European countries (Table 1). As with human isolates, these isolates predominantly belong to CC130 and to a lesser degree ST425. These lineages therefore appear to have a very broad host tropism. There are few data on the prevalence among animals, although a British study of bovine bulk tank milk found that 2.67% of dairy farms in England were positive for *mecC* MRSA but, interestingly, no positive farms were found in Scotland during the same survey [26]. Assessing the prevalence of *mecC* MRSA among different livestock species, understanding their role in veterinary disease, and the risk of zoonotic transmission are important topics for future research.

## Zoonotic potential of *mecC* MRSA

Both CC130 [27] and ST425 [6] have previously been regarded as animal-adapted lineages of *S*. *aureus*, suggesting that *mecC* MRSA arose in animals, possibly ruminants, and subsequently spread to humans [6]. Although the origins of *mecC* MRSA are not yet clear there is good evidence that contact with animals poses a zoonotic risk and that *mecC* MRSA can be transmitted between species – and therefore could be regarded as a LA-MRSA. For instance, most isolates in Denmark come from rural areas [19], and epidemiological follow-up of 22 patients found known animal contact in four cases [19]. Genome sequencing of *mecC* MRSA isolates from two of these cases provided compelling evidence of cross-species transmission with human and individual animal (cow and sheep) isolates being separated by only a few single-nucleotide polymorphisms across the entire core genome [28]. A survey of delegates at British Cattle Veterinary Association Congress in 2011 failed to find any *mecC* MRSA, providing evidence that the prevalence in this population is below 1% [29]. However, many cases of *mecC* MRSA do not have apparent animal contact, and household transmission between people has been demonstrated [30].

## *mecC* MRSA and disease in humans and animals

*mecC* MRSA have been isolated from carriage and a range of infections in humans (see Table 1 for list of references). These are predominantly skin and soft-tissue infections but include severe bone infections [31], nosocomial pneumonia [13] and fatal bacteraemia [30]. *mecC* MRSA can also cause disease in veterinary species; for example, mastitis in dairy cattle has been noted in several countries, and other examples include chronic conjunctivitis in a domestic cat [32] and a rabbit isolate of *mecC* MRSA from Belgium belonging to a highly virulent clone among farmed rabbits [33,34]. It appears that, similarly to conventional MRSA lineages, *mecC* MRSA strains are highly-versatile pathogens able to cause a wide range of infections in a range of host species, including severe and fatal infections. In agreement with these epidemiological observations, microarray analysis and genome sequencing reveal that *mecC* MRSA isolates encode several known or putative *S*. *aureus* virulence factors, including several adhesins, superantigens, and toxins [13,28,35]. In addition, a novel allele of *etd* (encoding exfoliative toxin D) with only 59% identify to the previously described *etd* gene was identified in CC130 isolates, and was putatively named *etd2* [28,36]. However, where tested, *mecC* MRSA strains have been negative for Panton–Valentine leukocidin [15,19], a prominent virulence factor among CA-MRSA, and they have been negative for the human immune evasion genes *sak*, *chp*, and *scn* [13,35], consistent with a possible origin for these strains in an animal reservoir.

Resistance to non-β-lactam antibiotics is currently uncommon among *mecC* MRSA isolates, and MICs for oxacillin and cefoxitin are generally low compared to those that can be seen among *mecA* MRSA. It will be of interest and of potential importance to monitor if these features change in the future.

## *mecC* in other species of staphylococci

The origins of *mecC* MRSA and SCC*mec* type XI are unclear, but *mecC* has also been detected by PCR in *Staphylococcus stepanovicii* from a wild European lynx in Austria [37], and a homologue, *mecC1*, located within a SCC*mec* IX-like element has been described using genome sequencing of *Staphylococcus xylosus* isolated from bovine milk in France [38] (Figure 1). This latter gene has 93.5% sequence identity to *mecC* in MRSA and is therefore classed as an allotype of *mecC* (≥70% but <95% nucleotide sequence identify) [7,38]. Finally, *mecC* has been found in *Staphylcoccus scirui* within a novel hybrid SCC*mec*–*mecC* element in isolates from caesarean incision wounds in Belgian Blue cattle [39] (Figure 1). As suggested for *mecA* [40,41], it is possible that *mecC* has its origin among coagulase-negative staphylococci; further investigations, including whole-genome sequencing of *mecC* staphylococci, may offer clues to the origin and evolution of this resistance determinant. These data also mean that clinical microbiology laboratories should be aware not only of *mecC* MRSA but also of the possible occurrence of *mecC* in other pathogenic species of methicillin-resistant staphylococci.

## Concluding remarks

*mecC* MRSA represent a recently recognised form of MRSA, encoding a divergent *mec* gene, which can colonise and cause disease in humans and a wide range of other host species. Although *mecC* MRSA are currently rare, and have only been reported in Europe to date, they present a potential diagnostic problem where there is reliance on *mecA* or PBP2a/2′ detection for MRSA diagnosis, and their emergence raises a several questions for future research (Box 1).

## Figures and Tables

**Table 1:** Distribution and characteristics of reported *mecC* MRSA

Country	Host species	Earliest reported isolate	*spa*-types	Multilocus sequence types (clonal complex)	Refs
UK	Human, dairy cattle, wild common seal, wild chaffinch, domestic dog	1993	t6300, t6292, t6220, t843, t6293, t1736, t1535, t7947, t7485, t7946, t7945, t6383, t742, t7734, t978, t6594, t7914, t9376, t6386, t9605, t8833, t11702, t11706, t9280	ST425 (CC425), ST130 (CC130), ST1245 (CC130), ST1526 (CC130), ST1944 (CC130), ST1764 (CC130), 1943 (CC1943/1946), ST1945 (CC130), ST1946 (CC1943/1946), ST2179 (CC599)	[6,15,21,26,33]
Denmark	Human, cattle, sheep	1975	t373, t528, t6220, t9397, t978, t2345, t3391, t8835, t9395, t843, t1535, t528, t1773, t1048, t3256, t1532, t1736, t3218, t3570, t5970, t9397, t5930 and t7603.	ST130 (CC130), ST1943 (CC130)	[6,33,42,43]
Republic of Ireland	Human	2010	t843 and t373	ST130 (CC130), ST1764 (CC130)	[9]
Germany	Human, wild hare, sheep, domestic dog, domestic cat, domestic guinea pig	2004	t843, t10513, t1736, 1773, t978, t7189, t1535, t10033, t10006, t1694, t278, t10009	ST130 (CC130), ST1945 (CC130), ST599 (CC599), ST2361 (CC1943/1946)	[13,20,37,44,45]
France	Human, dairy cattle	2007	t9280, t843	ST130 (CC130), ST1945 (CC130)	[31,46]
The Netherlands	Human	Not provided	Not provided	Not provided	[35]
Belgium	Wild brown rat, farmed rabbit, dairy cattle, beef cattle	1995	t208, t742, t9925, t1736	ST2273 (CC49), ST425 (CC425), ST2508 (CC599), ST130 (CC130)	[33,47]
Sweden	Dairy cattle, wild hedgehog	2003	t524, t9111	ST130 (CC130), ST425 (CC425)	[36,48]
Norway	Domestic cat	2012	t6902	ST2497 (CC1943/1946)	[32]
Austria	Wild European otter and wild European hedgehog	Winter 2012/13	t4335 and t3256	ST2620 (CC130), ST130 (CC130)	[37]
Spain	Human	2008	t843 and t6220	ST130 (CC130), ST1945 (CC130)	[30,49]
Switzerland	Human	2011	t11150	ST130 (CC130)	[22]
Finland	Dairy cattle	2006	t3256	ST130 (CC130)	[50]

## Box 1 Outstanding questions

• How widespread and common are *mecC* MRSA strains? To date they have only been reported in Western Europe, but are they present elsewhere, remaining undetected, or will they spread elsewhere?
• What are the cause(s) of the recent increase in prevalence reported from Denmark; will this continue and will this be seen elsewhere?
• How common are *mecC* MRSA strains among animal populations (wild, companion, and livestock), and how important are they for animal health or as a zoonotic reservoir for human MRSA infections?
• How and where did *mecC* and SCC*mec* type XI originate, and when and how often have they been acquired by *Staphylococcus aureus*? Why is CC130, and to a lesser degree ST425, so dominant among *mecC* MRSA isolates? Will it spread to other lineages, and will current *mecC* MRSA lineages acquire additional resistance determinants?
• What is the basis for the apparent broad host specificity shown by the *mecC* MRSA lineages CC130 and ST425?
• What is the structural and evolutionary basis for the functional differences between *mecA* and *mecC*-encoded PBP2a/2′?
• What are the best methods (e.g., chromogenic plates or antimicrobial susceptibility testing approaches) to detect *mecC* MRSA? Do diagnostic work-flows need to be altered to improve *mecC* MRSA detection?
• What is the distribution and prevalence of *mecC* among other staphylococci, and do these contribute to disease in animals and humans? Did they play a role in the origin of *mecC* and its dissemination to *S*. *aureus*?
